# Promoting Dementia‐Preventive Lifestyles in Aging Communities: A Motivation and Behavior Reinforcement Approach

**DOI:** 10.1002/alz70860_100852

**Published:** 2025-12-23

**Authors:** Hyunseo An, Hae Yean Park

**Affiliations:** ^1^ Graduate School, Yonsei University, Wonju, Wonju, Korea, Republic of (South); ^2^ College of Software and Digital Healthcare Convergence, Yonsei University, Wonju, Heungup‐meon, Korea, Republic of (South)

## Abstract

**Background:**

This study explored the community applicability of a dementia prevention lifestyle change motivation and behavior reinforcement program based on the Health Belief Model (HBM) among middle‐aged and older adults.

**Method:**

A one‐group pre‐post study was conducted with nine community‐dwelling middle‐aged and older adults. The 10‐session, 5‐week program incorporated the Knowledge, Evaluation, Experience, Plan (KEEP) strategy and a gold medal reward system to enhance motivation and reinforce dementia‐preventive behaviors. Assessments included the K‐MCLHB‐DRR, Dementia Preventive Behavior Scale, YLP‐BREF, PHQ‐9, and WHOQOL‐BREF. Wilcoxon signed‐rank tests analyzed pre‐ and post‐program differences, and satisfaction surveys and interviews were conducted. A 4‐week follow‐up assessed plan adherence.

**Result:**

Significant improvements were found in perceived benefits (*p* = .018) and self‐efficacy (*p* = .034) for dementia prevention motivation. Dementia‐preventive behaviors improved in cognitive (*p* = .016), social (*p* = .011), and health‐promoting activities (*p* = .027). Secondary outcomes showed enhancements in multifaceted lifestyle (*p* = .008), physical activity (*p* = .008), activity participation (*p* = .011), and depression (*p* = .018), with no significant change in quality of life. Participants reported high satisfaction and successful plan implementation at follow‐up.

**Conclusion:**

The HBM‐based program positively influenced dementia prevention motivation and behaviors, demonstrating its applicability within the community and providing a foundation for future interventions.

